# Sexual dimorphism of adipose tissue distribution across the lifespan: a cross-sectional whole-body magnetic resonance imaging study

**DOI:** 10.1186/1743-7075-6-17

**Published:** 2009-04-16

**Authors:** Wei Shen, Mark Punyanitya, Analiza M Silva, Jun Chen, Dympna Gallagher, Luís B Sardinha, David B Allison, Steven B Heymsfield

**Affiliations:** 1 Obesity Research Center, St. Luke's-Roosevelt Hospital & Institute of Human Nutrition, Columbia University, College of Physicians & Surgeons, New York, NY, USA; 2 Exercise & Health Laboratory, Faculty of Human Movement, Technical University of Lisbon, Lisbon, Portugal; 3 Department of Biostatistics, University of Alabama at Birmingham, Birmingham, AL, USA; 4 Merck & Co, Rahway, NJ, USA

## Abstract

**Background:**

Despite increasing research and clinical significance, limited information is available on how the visceral and subcutaneous adipose tissue (VAT and SAT) compartments develop during growth and maturation and then vary in volume across the adult lifespan. The present study aimed at exploring how adipose tissue compartments partition across the lifespan.

**Methods:**

Total body VAT and SAT were quantified in an ethnically-diverse cross-sectional sample of healthy subjects ages 5 – 88 yrs [children (5–17 years): males n = 88, BMI percentile (X ± SD), 61.9 ± 27.1; females, n = 59, BMI percentile, 60.0 ± 28.4; adults (≥ 18 yrs): males, n = 164, BMI, 25.6 ± 3.7 kg/m^2^, and females, n = 188, BMI, 25.5 ± 5.4 kg/m^2^]. Subjects completed a whole-body magnetic resonance imaging scan and images were then segmented for VAT and SAT; total compartment volumes were calculated from respective slice areas. Sex and age distributions were evaluated by generating quadratic and cubic smoothing lines fitted to the data. Plots were developed with and without adjustment for total adipose tissue, ethnicity, and menopausal status in women. VAT and SAT volumes were both larger with greater age.

**Results:**

In adulthood, VAT was larger in males than in females with and without adjustment. In contrast, SAT volume was larger in females than in males after entering puberty and sex differences remained, with and without adjustment, across the remaining lifespan.

**Conclusion:**

Based on observations made in this cross-sectional sample, VAT and SAT volumes were variably larger with greater age across most of the human lifespan, although the relatively small number of children warrants future larger scale studies to validate our observations. Moreover, the pattern and magnitude of adipose tissue "growth" differed between males and females, with the mechanistic basis of this sexual dimorphism only partially understood. These descriptive observations in a large cross-sectional cohort provide an initial foundation for future longitudinal and cohort studies.

## Background

There is growing recognition that adipose tissue depots vary widely in their metabolic properties [1,2]. A related observation is a strong association between selected adipose tissue compartments and the health risks of obesity [1,3,4]. Visceral adipose tissue (VAT), particularly the portion located in the mesenteric and omental areas, is associated with the risk of developing cardiovascular disease and type II diabetes [5,6]. This relatively small compartment, less than 5% of body mass in young non-obese adults, is an important component of the metabolic syndrome and other conditions related to insulin resistance [6,7]. The presence of excess VAT also increases the risk for developing gastroesophageal reflux [8], cholesterol gallstones [9], sleep apnea [10], Alzheimer's disease [11], and other chronic medical conditions. In rodents, surgical removal of VAT leads to an improved metabolic profile [12] and increased longevity [13].

Measuring VAT accurately in living humans remains challenging. VAT is difficult to quantify separately from subcutaneous adipose tissue (SAT) [14]. Accordingly, limited information is available on how VAT and SAT compartments develop during growth and maturation and vary across the adult years. Existing information is fragmentary, leaving important gaps in our understanding of adipose tissue partitioning across the lifespan. This information could prove useful in the understanding of conditions that are associated with excess VAT and total adiposity [15].

To fill an important gap in the current literature, we examined VAT and SAT volumes in relation to each other and total body adipose tissue (TAT) using compartment estimates derived from whole-body magnetic resonance imaging (MRI) studies in a cross-sectional sample of healthy subjects between the ages of 5 and 88 years. The results support and extend limited earlier studies demonstrating a clear sexual dimorphism in the development and growth of the VAT and SAT compartments that persist throughout life.

## Methods

### Experimental Design

A cross-sectional subject sample was evaluated over a ten year period as part of research programs investigating body composition and related metabolic disorders at the New York Obesity Research Center. Whole-body estimates of VAT, SAT, and TAT were used to generate sex-specific age plots that were then evaluated with or without adjustment for potential influencing factors.

### Subjects

Each subject was screened with a medical history, physical examination, and blood studies. Subjects were retained in the current database who were healthy, free of acute and serious chronic diseases, and who were not engaged in high level physical activity training programs. Subjects weighing more than 300 pounds (136 kg) were excluded from the study due to the MRI system weight limit. There were no race restrictions and race was established by self-report. A lower age limit of 5 years was set as children below this threshold are usually unable to comfortably tolerate the imaging procedure. Once medically cleared, each subject completed the whole-body MRI scan for measurement of adipose tissue volumes.

Body weight was measured to the nearest 0.1 kg and height to the nearest 0.1 cm using appropriately calibrated scales and stadiometers.

### Magnetic Resonance Imaging

Whole-body MRI scans were acquired, as previously reported by our group [16,17], using a 1.5 T General Electric system (6X Horizon, Milwaukee, WI). Seventy-one children (ages 5 to 17 years) and all 352 adults (age ≥ 18 years) were scanned by protocol 1, which acquires cross-sectional images of 10 mm thickness at 40 mm intervals from fingers to toes with the subject in either a prone or supine position, resulting ~40 images in each subject. The L_4_-L_5 _inter-vertebral disc was set as the point of origin. A subset of 76 children was scanned by protocol 2, which acquires cross-sectional images of 10 mm thickness without any gaps, resulting ~200 images in each subject. In each subject scanned with protocol 2, a subset of images was selected by including every slice and by skipping every four slices. The selection of this subset of images from protocol 2 includes ~40 images, with an image slice interval and thickness that exactly matches that of protocol 1. As this subset of images from protocol 2 was used for data generation, all subjects can be considered as using the same scanning protocol (i.e., protocol 1). Following acquisition, VAT and SAT were segmented at the New York Image Reading Center by trained and quality-controlled technicians using image analysis software (SliceOmatic, Tomovision Inc., Montreal, Canada). Visceral adipose tissue volumes were calculated using all slices between the dome of the liver and the bottom of the pelvis (abdominopelvic region). Adipose tissue compartment volume (*V*) was calculated as:



where *A*_*i *_is each scan's cross-sectional area, *h *is the between-slice interval, *t *is the thickness of each slice, and *N *is the number of total slices. The intra-class correlation coefficient based on 2-way mixed effects model for volume rendering of SAT and VAT for the same scan by different analysts are 0.99, and 0.95, respectively [18].

### Statistical Methods

Group data are presented as the mean ± SD for children (5–17 years) and adults (age ≥ 18 years). Visceral adipose tissue and SAT volumes were plotted against age for males and females separately.

Regression models were used to investigate the relationships between age and adipose tissue compartments. Nonlinear effects of age on VAT and SAT were tested by including quadratic and cubic terms and their interactions with sex. Covariates making significant contributions were retained in the model. Regression analyses were also conducted for VAT and SAT volumes after adjustment for TAT, ethnicity, and menopause status for females.

Cubic or quadratic lines, depending on the final regression model for VAT and SAT with and without adjustment, were then fitted separately for males and females for the age range of 2.5 to 97.5 percentile (i.e., age 7 to 78 years).

Regression analyses were also conducted separately for pre- and post- menopausal women, and for prepubertal and pubertal children. The intra-class correlation coefficient was calculated with subjects as a random effect and MRI analyst as a fixed effect based on a 2-way mixed effects model (i.e., model 3, 1) [18].

All statistical analyses were carried out using SPSS (SPSS for Windows, 16.0, SPSS Inc., Chicago, USA) and we used two-tailed (α = 0.05) tests of significance.

## Results

### Subject Characteristics

The subject demographic and body composition data are presented in Table 1. The multiethnic group included 147 children (ages 5 – 17 years; n = 88 males and 59 females) and 352 adults (age ≥ 18 years; n = 164 males and 188 females). The adult and child groups had mean BMIs or BMI percentiles approximating those observed in the general US population [19,20]. Among the adults, males were younger than females (P < 0.001) and there were no sex differences in BMI. Age and BMI percentiles in the child group did not differ significantly between males and females.

**Table 1:** Subject Characteristics.

	***Adults*^†^**			***Children*^†^**		
	**Males**	**Females**	**P value**	**Males**	**Females**	**P value**

**Total Subjects**	164	188	N/A	88	59	N/A
**Caucasian**	67	84	N/A	13	9	N/A
**African American**	38	53	N/A	33	28	N/A
**Hispanic**	27	24	N/A	29	19	N/A
**Asian**^‡^	28	26	N/A	3	0	N/A
**Age (yrs)**	37.8 ± 14.8 (35.0, 27.0, 44.0)	44.0 ± 17.4 (41.0, 30.0, 56.0)	<0.001	11.7 ± 3.6 (11.0, 9.0, 15.0)	11.3 ± 3.4 (11.0, 8.0, 14.0)	0.635
**Weight (kg)**	80.2 ± 13.0 (79.5, 71.3, 88.3)	67.1 ± 14.9 (65.0, 54.7, 77.6)	< 0.001	50.9 ± 21.7 (50.0, 31.3, 65.1)	47.7 ± 18.5 (47.2, 33.1, 58.3)	0.504
**BMI (kg/m^2^)**	25.6 ± 3.7 (25.1, 22.5, 28.3)	25.5 ± 5.4 (24.7, 21.5, 28.9)	< 0.001	20.6 ± 4.6 (19.9, 16.9, 23.1)	20.9 ± 5.3 (20.3, 17.1, 23.1)	0.441
**BMI percentile**	N/A	N/A	N/A	61.9 ± 27.1 (71.2, 48.7, 92.8)	60.0 ± 28.4 (72.8, 46.5, 95.5)	0.827
**TAT (L)**	18.8 ± 8.1 (17.3, 12.4, 23.2)	25.2 ± 11.9 (21.9, 15.9, 32.2)	< 0.001	13.4 ± 9.5 (10.2, 6.8, 16.8)	17.7 ± 12.5 (14.0, 8.8, 22.6)	0.020
**VAT (L)**	2.1 ± 1.8 (1.5, 0.7, 3.0)	1.4 ± 1.2 (1.0, 0.6, 2.1)	< 0.001	0.6 ± 0.5 (0.4, 0.2, 0.8)	0.6 ± 0.5 (0.5, 0.3, 0.8)	0.762
**SAT (L)**	16.5 ± 6.9 (15.6, 11.3, 20.2)	23.7 ± 11.1 (20.8, 14.9, 30.5)	< 0.001	12.2 ± 8.5 (9.3, 6.2, 15.3)	16.4 ± 11.6 (13.0, 8.0, 21.1)	0.013

Results are expressed as mean ± SD and (median, 25^th ^and 75^th ^percentile). ^†^, Children are ages 5–17 years and adults ≥ 18 years. ^‡^, the Asian sample is a multi-generation mixture of Chinese, Indian, Korean, and Japanese. Abbreviations, N/A, not applicable; TAT, SAT, and VAT are total, subcutaneous, and visceral adipose tissue.

### Adipose Tissue Components

Total adipose tissue was significantly smaller in the adult males than in the females (18.8 ± 8.1 L vs. 25.2 ± 11.9 L; p < 0.001). TAT was also significantly smaller in the boys than in the girls (13.4 ± 9.5 L vs. 17.7 ± 12.5 L; p = 0.020).

VAT was significantly greater in the adult males than in the females (2.1 ± 1.8 L vs. 1.4 ± 1.2 L; p < 0.001). There were no sex differences in VAT in the children (0.6 ± 0.5 vs. 0.6 ± 0.5 L; p = 0.762).

SAT volume was significantly smaller in the adult males than in the females (16.5 ± 6.9 L vs. 23.7 ± 11.1 L; p < 0.001) and significantly smaller in the boys than in the girls (12.2 ± 8.5 vs. 16.4 ± 11.6 L; p = 0.013).

### Age and Sex Effects

**Figure 1:**
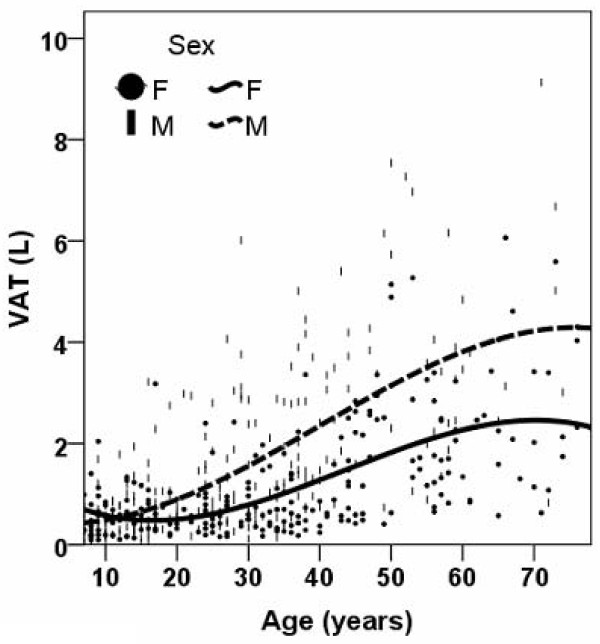
**Smoothed scatter plots for visceral adipose tissue (VAT) volumes versus age for males and females**.

**Figure 2:**
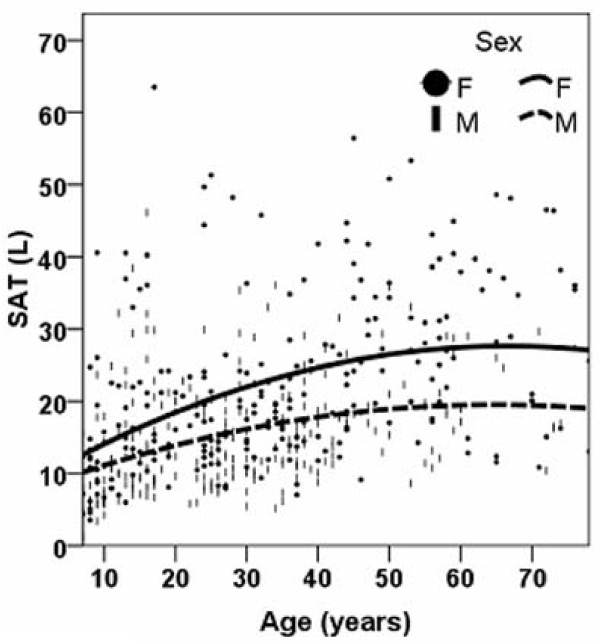
**Smoothed scatter plots for subcutaneous adipose tissue (SAT) volumes versus age for males and females**.

The regression equations for each adipose tissue component are presented in Table 2. The smoother line for VAT and SAT volumes versus age are shown in Figure 1 and Figure 2, respectively. The figure shows that VAT and SAT are both larger with greater age across the entire age range except for a reverse trend before age 17 years in females (i.e., VAT at age 7, 0.70 L, VAT age 17, 0.48 L). Males had a larger amount of VAT than females after age 12 years and this difference remained throughout the remaining age range (Figure 1). In contrast, females had a larger SAT volume than males across the whole age range (Figure 2). In both males and females, the slope of the regression line between SAT and age was smaller for subjects over the age of 50 years older than for their younger counterparts (slopes in males, 0.003 vs 0.204; slopes in females, 0.021 vs 0.322).

**Figure 3:**
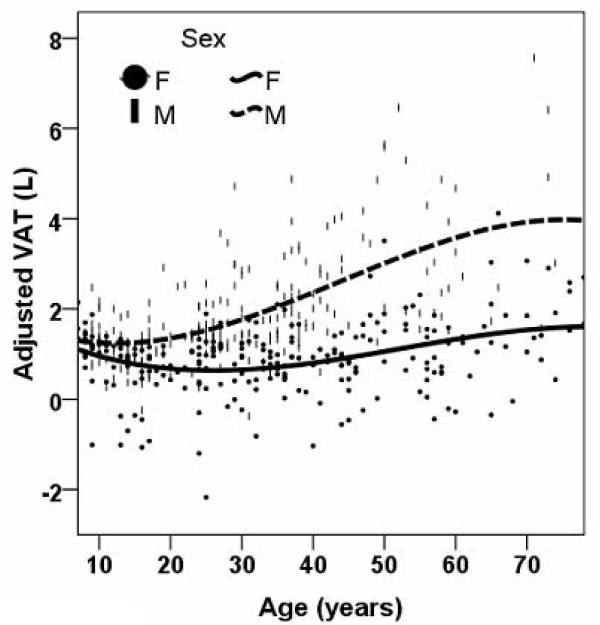
**Smoothed scatter plots for visceral adipose tissue (VAT) volumes versus age for males and females after adjustment for total adipose tissue volume, ethnicity and menopause status in females**.

**Figure 4:**
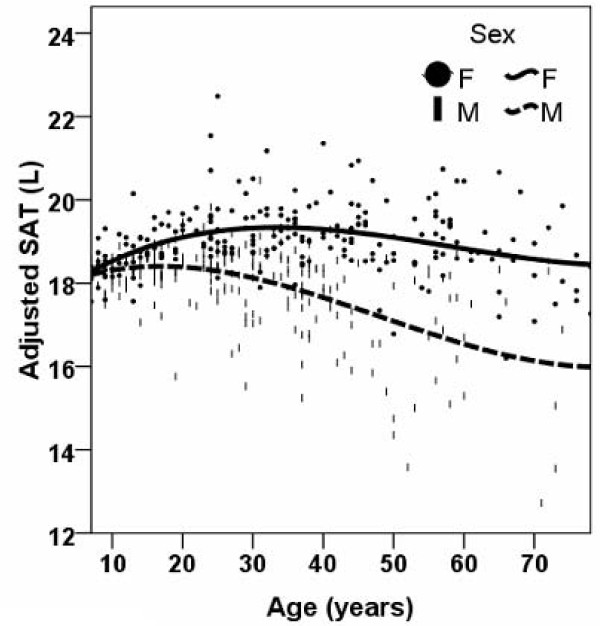
**Smoothed scatter plots for subcutaneous adipose tissue (SAT) volumes versus age for males and females after adjustment for total adipose tissue volume, ethnicity and menopause status in females**.

After adjustment for TAT volume and ethnicity, in males VAT was smaller with greater age before the age of 12 years and VAT was larger after the age of 12 years throughout the remaining age range (Figure 3). After adjustment for TAT, ethnicity, and menopause status in females, VAT was smaller with greater age before the age of 26 years and larger after the age of 26 years throughout the remaining age range (Figure 3). After adjustment for TAT volume and ethnicity, SAT was larger for older age before the age of 17 years in males and smaller after the age of 17 years throughout the remaining age range (Figure 4). In females SAT was larger with increasing age before the age of 35 years and SAT was smaller with increasing age after the age of 35 years (Figure 4).

**Table 2:** Regression equations for adipose tissue compartments (n = 499).

	Estimate of regression coefficients for independent variables								Adjusted R^2^
			
	Age (year)	Age^2^	Age^3^	Sex	Age × sex	Age^2 ^× sex	Age^3 ^× sex	Intercept	
VAT	-2.22 × 10^-2^	2.59 × 10^-3^	-2.15 × 10^-5^	6.97 × 10^-1^	-6.77 × 10^-2^	7.52 × 10^-4^	-4.10 × 10^-6^	4.73 × 10^-1^	0.428
SAT	4.47 × 10^-1^	-3.26 × 10^-3^		5.54 × 10^1^				5.88 × 10^1^	0.244
Adjusted VAT	-6.10 × 10^-2^	2.89 × 10^-3^	-2.20 × 10^-5^	-1.76 × 10^-2^	-2.01 × 10^-2^	-8.11 × 10^-4^	8.77 × 10^-6^	1.59 × 10^0^	0.440
Adjusted SAT	7.89 × 10^-2^	-2.84 × 10^-3^	1.97 × 10^-5^	-2.62 × 10^-1^	4.67 × 10^-2^	-2.02 × 10^-4^	-4.52 × 10^-6^	1.78 × 10^1^	0.431

VAT, visceral adipose tissue, SAT, subcutaneous adipose tissue. Sex, Female = 1, Male = 0. All R^2 ^are significant at P < 0.05

### Menopause Effects

When regression analyses for both VAT and SAT were completed in pre- and post- menopausal women separately, age^2 ^(P = 0.003, P < 0.001 for VAT and SAT respectively) and age^3 ^(P = 0.001, P < 0.001 for VAT and SAT respectively) significantly entered the model in pre-menopausal women. However, there were non-significant relationships between age and SAT (P = 0.130) and age and VAT (P = 0.872) in postmenopausal women. When pre- and post- menopausal women were pooled, menopausal status was no longer significant for both VAT and SAT, either as an individual term (VAT, P = 0.279; SAT, P = 0.272) or as interactions with age (VAT, P = 0.284; SAT, P = 0.440).

The increase of VAT was calculated for every age decade. In women, the VAT increases from ages 40 to 50 years (i.e., mean volume, 0.55 L) and from age 50 to 60 years (i.e., mean volume, 0.45 L) were the largest observed.

### Puberty Effects

There were a total of 115 children (i.e., ~78%) with pubertal status available. One 16 year old child with Tanner Stage 1 was excluded from the data analysis as an outlier. When regression analyses for both VAT and SAT were completed in pre-pubertal and pubertal children separately, age did not significantly (P = 0.495, P = 0.337 for VAT and SAT, respectively) enter the model in pre-pubertal children when controlled for sex (Table 3). On the other hand, age borderline significantly or significantly (P = 0.068, P = 0.004 for VAT and SAT, respectively) entered the model in pubertal children when controlled for sex (Table 3). Sex did not significantly enter the models in prepubertal children for VAT (P = 0.235), and SAT (P = 0.259). In pubertal children, sex was significant in the SAT model (P = 0.004) but not in the VAT model (P = 0.722). When pre-pubertal and pubertal children were pooled, age significantly entered the model for VAT (P = 0.021) and SAT (P = 0.001). In the pooled data, pubertal status was not significant for both VAT and SAT, either as an individual term (VAT, P = 0.907; SAT, P = 0.718) or as interactions with age (VAT, P = 0.802; SAT, P = 0.415).

**Table 3:** Regression equations for adipose tissue compartments in prepubertal and pubertal children.

	Prepubertal				Pubertal			
	
	Age	Sex	Intercept	Adjusted R^2^	Age	Sex	Intercept	Adjusted R^2^
VAT	0.029 (P = 0.495)	0.128 (P = 0.235)	0.102 (P = 0.604)	0.001 (P = 0.374)	0.050 (P = 0.068)	0.051 (P = 0.722)	- 0.055 (P = 0.938)	0.020 (P = 0.187)
SAT	0.658 (P = 0.337)	1.962 (P = 0.259)	2.437 (P = 0.509)	0.010 (P = 0.317)	1.329 (P = 0.004)	7.100 (P = 0.004)	- 5.200 (P = 0.426)	0.157 (P = 0.001)

VAT, visceral adipose tissue, SAT, subcutaneous adipose tissue. Sex, Female = 1, Male = 0.

## Discussion

The increasing focus on conditions such as "visceral adiposity" and "central obesity" (1–12) prompted the examination for the first time in a cross-sectional cohort total body VAT and related SAT volumes across most of the human lifespan. Our study applied whole body MRI measurements to describe these adipose tissue relations in a large and diverse sample. Compared to previous studies using a single image slice or a few aggregated image slices at the abdomen, the whole body MRI technique applied in the present study has the advantage of measuring whole body SAT and total abdominopelvic VAT. Compared to CT, MRI does not expose the subject to radiation and therefore has the advantage of providing volumetric measurement. VAT and SAT volumes were both larger with greater age with "growth" of these two compartments not stopping after childhood but continuing at variable rates across most of the adult lifespan. VAT was larger in males than in females after puberty and this sex difference remained across the remaining lifespan. In contrast, SAT was greater in females than in males across the lifespan, revealing clear sex differences in adipose tissue partitioning.

### Adipose Tissue Partitioning

#### Sex and Age Effects

Our results show that VAT was larger with greater age across the evaluated lifespan, which agrees with early small scale studies using multi- or single slice CT scan measurements [21,22] as well as whole body MRI scans in adult Caucasians at increased risk of developing type 2 diabetes [23]. Several studies have reported that the age-related increase in VAT is greater in males than in females using methods ranging from single slice CT to whole body MRI [21,23,24].

We found that after adjusting for TAT, ethnicity, and menopause status in females, there was greater VAT and less SAT with advancing age after approximately age 30 years. This reflects that although VAT and SAT both increase with age, the proportion of VAT as total adipose tissue increases with age and the proportion of SAT as total adipose tissue decreases with age. Our results also agree with Zamboni et al.'s findings that age-associated changes in abdominal adipose tissue distribution are not always reflected by a change in TAT [22]. In one longitudinal study of 65 women who were annually evaluated for up to 4 years, the increase in VAT was greater over time than the increase in TAT [25]. This agrees with our results of the relatively small slope between SAT and age after age 50 years.

The smaller slope of VAT or SAT present at older ages (i.e., 50 years and older) than that of their younger counterparts (slopes in males, 0.003 vs 0.204; slopes in females, 0.021 vs 0.322)) (Figure 1 and 2) may reflect a survival advantage of non-obese individuals and the exclusion of obese subjects who had co-morbid conditions since we only included healthy subjects in our study.

#### Potential Pubertal Effects

Our results in Table 1 show no difference in the amount of VAT in 5–17 year old children; further analysis by regression modeling confirmed the absence of a sex difference in VAT in either prepubertal or pubertal children (Table 3). We observed a significant sex difference in SAT amount (Table 1) and regression modeling in children revealed that this sex difference only exists in pubertal children but not prepubertal children (Table 3). Our results also show that the larger amount of VAT or SAT with older age may present in pubertal children but not prepubertal children. Of note, although Figure 1 depicts well the overall trend of the relationship between age and adipose tissue components across the whole life span, the results based on regression analysis of the children's data (Table 3) provides more insight into this relationship specifically in childhood and puberty. Our results agree with Fox et al's [26] finding that excess fat storage is initially greater in the subcutaneous compartment than in the visceral compartment. Both Benfield et al and Gutin et al.'s studies also showed that VAT remain relatively small in children [27,28]. Although it is well established that males have more VAT than females, our findings suggest that there is no sex difference in the amount of VAT in children. Gutin et al. found black girls have more VAT than black boys but white girls have similar amount of VAT as white boys [27]. Benfield et al. showed that girls between the age of 12 and14 years have more VAT than boys of the same age range [28]. The difference between our results and previous studies can be potentially attributed to 1) ethnic differences of samples, and 2) population [28] vs. non-population studies [27].

An earlier longitudinal study of children between the ages of 5 and 12 years showed that the VAT compartment increased significantly over a 5 year follow up period [29]. However, Brambilla et al. found that there is a tendency for VAT to decrease in normal weight children and remain stable in obese children during puberty [30,31]. Since puberty may have an independent effect on adipose tissue distribution [32,33] and Brambilla et al.'s studies only included peri-pubertal children, it is possible that pubertal stage contributes to the inconsistencies across these studies. Puberty may also influence the way in which changes in adipose tissue distribution occurs between lean and obese children.

As puberty stage data were unavailable for all children in the present study, data analysis was only conducted in prepubertal and pubertal children. Further investigation is needed to clarify the relationship between age, growth, and pubertal stage with a longitudinal design starting before puberty onset and lasting until the post pubertal period.

#### Menopausal Effects

Several studies have shown a preferential accumulation of VAT in post-menopausal women [21,22,34,35]. These body composition changes may in part be due to alterations in sex hormone concentrations that are associated with menopause [36,37]. Indeed, ovarian hormone deficient women have a significantly higher lipoprotein lipase activity measured by the rate of fatty acid uptake in the VAT depot than do pre-menopausal women [37]. Furthermore, hormone replacement therapy may attenuate the propensity to increase VAT [38]. Together, these reports support our findings of an enlarged VAT compartment between the ages of 40 and 50 years and ages of 50 and 60 years. Our results also suggest that the larger VAT volume with greater age occurs not only in post-menopausal women, but also within the 10 year period before the onset of menopause. This is in accordance with the observation that the peri-menopausal period is associated with changes in the hormonal milieu [39] and also accumulation of central fat [40].

Armellini and colleagues [41] reported that intra-abdominal adipose tissue negatively correlates with serum testosterone levels in obese women. However, prospective data in post-menopausal women show no association between measured bioavailable testosterone or the ratio of testosterone to sex hormone binding globulin and the ratio of waist to hip circumference [42]. These findings do not support the theory that androgens cause visceral adiposity in post-menopausal women.

### Study Limitations and Future Directions

There are several limitations of this study. First, we cannot exclude the possibility of the existence of a subject selection bias. Although ideally our relatively large and ethnically diverse sample along with controlling for ethnicity minimize selection bias, individuals who continued to increase abdominal adiposity throughout their life span may have developed diseases and would have been excluded from participation in this study because we only included healthy subjects. As the prevalence of obesity has increased over the past two decades the possibility also exists that younger subjects are more obese than older subjects and therefore the cross-sectional results from the study may be different from what would have been observed had we conducted a longitudinal study. Similarly, the increase in the prevalence of obesity may also induce changes in adipose tissue distribution if adipose tissue distribution is a function of total adiposity. This possibility implies that younger subjects' adipose tissue distribution may be different from older subjects' adipose tissue distribution secondary to greater adiposity in the younger subjects. However, a recent study showed that the relationship between BMI and waist circumference as characterized by the slope of the linear regression of waist circumference on BMI does not appear to be changing appreciably over time [43]. This implies that fat distribution may not change with total adiposity at a population level.

Second, we did not control for cardiorespiratory fitness, which is also related to visceral adiposity [35,44]. Our study was confined to non-athletes and measures of cardiorespiratory fitness were unavailable to us.

Third, we did not control for hormone replacement therapy that may affect abdominal adiposity.

Fourth, compared to most epidemiological studies, the scale of our study is relatively small (i.e., N = 499). Our results should therefore not be used as a population reference data.

Finally, there are race differences in adipose tissue distribution [45]. Although we controlled for race differences in this study, we did not have enough subjects in each ethnic group to examine differences in adipose tissue partitioning differences across the whole life span.

Despite the above mentioned limitations, our study is unique in depicting the preliminary trends in the relationships between adipose tissue partitioning and age based on a diverse cross-sectional sample. The results generated from the present study can be used to guide future longitudinal studies adipose tissue growth and its change with aging.

## Conclusion

Based on observations made in this cross-sectional sample, VAT and SAT volumes were variably larger with greater age across most of the human lifespan, although the relatively small number of children warrants future larger scale studies to validate our observations. Moreover, the pattern and magnitude of adipose tissue "growth" differed between males and females, with the mechanistic basis of this sexual dimorphism only partially understood. These descriptive observations in a large cross-sectional cohort provide an initial foundation for future longitudinal and cohort studies.

## List of abbreviations

MRI: magnetic resonance imaging; SAT: Subcutaneous adipose tissue; TAT: Total adipose tissue; VAT: Visceral adipose tissue.

## Competing interests

The authors declare that they have no competing interests.

## Authors' contributions

WS and SBH designed the study. WS, MP and JC organized data. WS analyzed the data. DBA provided critical statistical advice. WS, AMS and SBH wrote the manuscript. MP, JC, LBS and DG provided advice. All authors read and approved the final manuscript.
